# Transactions of the Kentucky State Medical Society, 1853

**Published:** 1853-05

**Authors:** 


*Transactions of the Kentucky State Medical Society*, 1853.—The Transactions of the Kentucky State Medical Society at the meeting held at Louisville, in October, 1852, have recently been published in a neat volume of three hundred and thirty-three pages. First, is an interesting address by the president, Dr. W. L. Sutton. Next comes the report of the standing committee on vital statistics, Dr. W. S. Chipley, chairman. In illustration of the advantages of a system of registration of deaths, the statistics of mortality for the city of Lexington during the last twenty-one years are given. On comparison, these show a progressively increasing ratio of deaths, a fact which proves the existence of causes of disease to a greater extent than heretofore. The practical consideration is, that these causes are, if possible, to be discovered and obviated — but these would probably have remained unsuspected save for the statistics which developed the knowledge of their existence.

The importance of the subject with reference to sanitary improvements, is earnestly and ably presented in this report.

Next follows a report on medical ethics, by Drs. Ashbury Evans and Wm. R. Chew.

The report on obstetrics is the next paper. This is from the pen of Prof. Miller, and a considerable portion of it appeared in our last issue.

A committee on registration report an act which, mainly through the efforts of its members, has been passed by the legislature of the state.

The report on surgery, by Prof. Gross, is the most elaborate paper contained in the Transactions. We have already taken occasion to notice this production, having had the pleasure of being present when it was read by its accomplished author. It is an historical account of the surgery of Kentucky. It is a comprehensive and complete history. Prof. Gross spared no pains in collecting information from all available sources, and he has rendered a most valuable service to the profession of the state. It is a work for the future, as well as the present; and it will remain, and be referred to in all time, as authority for the historical details which it embraces. We have already referred to the biographical sketch of Dr. McDowell, the author of the operation of ovariotomy. This portion of the report will be read with great interest.

Succeeding the paper by Prof. Gross, is a report on indigenous botany, accompanied by a catalogue of the plants of Kentucky, from the pen of Dr. C. H. Spilman.

A brief report on epidemics follows, by Drs. Darby and D. P. Drake.

In conclusion, a committee, consisting of Drs. T. S. Bell, E. D. Foree, and

H. S. Chipley, report a plan for a case, book which, under the auspices of the society, has since been published.

Judging from such a beginning, this being the first volume of Transactions issued, the society has entered on a career of honor and usefulness.

